# Genotyping and phylogenetic analysis of *Coxiella burnetii* in domestic ruminant and clinical samples in Iran: insights into Q fever epidemiology

**DOI:** 10.1038/s41598-023-47920-0

**Published:** 2023-11-21

**Authors:** Ashraf Mohabati Mobarez, Neda Baseri, Mohammad Khalili, Ehsan Mostafavi, Saber Esmaeili

**Affiliations:** 1 Department of Bacteriology, Faculty of Medical Sciences, Tarbiat Modares University, Tehran, Iran; 2 Department of Epidemiology and Biostatics, Pasteur Institute of Iran, Tehran, Iran; 3 National Reference Laboratory for Plague, Tularemia and Q Fever, Research Centre for Emerging and Reemerging Infectious Disease, Pasteur Institute of Iran, Akanlu, Kabudar-Ahang, Hamadan Iran; 4 Department of Pathobiology, Faculty of Veterinary Medicine, Shahid Bahonar University of Kerman, Kerman, Iran

**Keywords:** Microbiology, Bacteria, Microbial genetics

## Abstract

*Coxiella burnetii*, a zoonotic pathogen, is the causative agent of Q fever, an endemic disease in Iran. However, there is currently a lack of available data on the genotypes of *C. burnetii* in the country. Here, we typed 26 *C. burnetii* isolates detected in milk, abortion, cotylodon, and cardiac valve samples from various geographical areas and hosts (7 cattle, 8 goats, 10 sheep, and 1 human) using Multilocus Variable Number Tandem Repeat Analysis (MLVA/VNTR) with five loci:ms24, ms27, ms28, ms33, and ms34. As IS1111 was observed to be spontaneously inserted in locus ms23 across all of our examined *C. burnetii* samples, five loci were employed for MLVA/VNTR genotyping. Among the 26 *C. burnetii* strains, 22 distinct genotypes (A–V) were identified in the discriminative loci. In silico analysis categorized Iranian *C. burnetii* strains into five genomic groups along with seven singletons, representing 11 exiting clonal complexes worldwide. Clusters 10 and 11 exclusively consisted of Iranian samples. These findings revealed high genotyping diversity among *C. burnetii* isolates in Iran. The genotypes circulating in Iran differed significantly from those found in other regions worldwide. To gain a comprehensive understanding of Q fever epidemiology in Iran, it is crucial to conduct large-scale studies that assess the distribution of *C. burnetii* genotypes across different geographical areas, hosts, and sources.

## Introduction

Q fever is a zoonotic disease caused by *Coxiella burnetii*, an obligate intracellular bacterium. While domestic ruminants, such as cattle, sheep, and goats, are the main reservoirs of *C. burnetii*, infections in these animals may be asymptomatic^1^. However, during outbreaks, goats may experience significant abortion rates of up to 90%^2^. The primary transmission route of *C. burnetii* to humans is through contaminated aerosols and dust particles. However, rare cases of transmission of infection to humans through ingestion of raw milk or dairy products, sexual contact, and blood transfusion have also been reported^3,4^.

Q fever can manifest in humans as either asymptomatic or self-limiting^5^, with acute cases resembling flu-like symptoms. In some instances, clinical manifestations of acute Q fever may include hepatitis and atypical pneumonia^6^, while chronic Q fever can lead to life-threatening endocarditis^3^.

Annually, there are reports of infections caused by *C. burnetii* in humans and animals worldwide^3^. In Europe, seroprevalence studies estimate the occurrence of *C. burnetii* in humans to be between 2 and 10%^7^. According to a meta-analysis study conducted in Iran in 2017, the overall seroprevalence of phase I and II IgG antibodies in humans was found to be 19.8% and 32.86%, respectively^1^. The prevalence of acute Q fever among suspected patients in northern and northwestern Iran was reported to be 13.8% and 5.37% in 2017 and 2019, respectively^8,9^. Q fever infective endocarditis was diagnosed in 30.77% of patients with culture-negative endocarditis in Iran from 2016 to 2018^10^. In addition, there have been several reports of *C. burnetii* infections in domestic livestock, wildlife, ticks, milk, and dairy product^1,11,12^. However, limited data exists on *C.* *burnetii* molecular typing in Iran. A recent study showed the circulation of five *C. burnetii* genotypes among Iranian domestic ruminants using the multi-spacer sequence typing (MST) method^13^.

Given the wide geographical distribution, diverse reservoirs, transmission routes, and clinical features of *C. burnetii*, studying its epidemiology presents a complex challenge. Therefore, molecular characterization of *C. burnetii* strains can provide valuable epidemiological data^14–16^. Comparative analysis of *C. burnetii* genotypes can help identify sources of infection in humans and animals, classify circulating strains, and contribute to infection control and prevention^17^. Multilocus Variable Number Tandem Repeat Analysis (MLVA/VNTR) genotyping, based on Variable Number of Tandem Repeats (VNTR), offers a helpful approach for *C. burnetii* genotyping. This method was first designed in 2006 for typing *C.* *burnetii* isolates, resulting in the identification of 36 different genotypes among 42 *C. burnetii* isolates^14^. The first panel of MLVA/VNTR genotyping contains eleven loci with repeat units equal to or longer than nine base pairs (bps), while the second panel includes six loci with repeat units of six or seven base pairs (bps) that require more advanced separation methods such as capillary electrophoresis^14,18^.

As Q fever is endemic in Iran and comprehensive data on genotyping of circulating *C. burnetii* strains is lacking, molecular genotyping using the MLVA/VNTR method can significantly contribute to understanding the molecular epidemiology of Q fever in Iran. Therefore, the present study aimed to address this gap by conducting molecular genotyping of selected *C. burnetii* isolates in Iran using the MLVA/VNTR method with the second panel.

## Material and methods

### Samples

The 26 *C. burnetii*-positive samples previously identified in Iran^10,19–21^ were selected for genotyping using the Dutch six-locus MLVA panel^22^. These positive samples consisted of one human sample (cardiac valve from a Q fever endocarditis case) and 25 animal samples. All samples included in this study were confirmed using a quantitative real-time PCR (qPCR) assay targeting IS1111 of *C. burnetii*^23^*.* The characteristics of included samples and their quantification cycle (Cq) values obtained via qPCR are summarized in Table 1.

**Table 1:** The characteristics of the *Coxiella burnetii*-positive samples included in the present study.

Sample	Reservoir	Source	Geographic origin	Cq* in real-time PCR
MB5	Cattle	Milk	Qom	31.55
MB14	Cattle	Milk	Qom	27.32
MB47	Cattle	Milk	Qom	25.24
MB92	Cattle	Milk	Tehran	29.61
MB98	Cattle	Milk	Tehran	27.25
AC3	Cattle	Spleen aborted fetus	Tehran	29.02
AC11	Cattle	Spleen aborted fetus	Tehran	27.82
MG96	Goat	Milk	Qom	32.27
MG101	Goat	Milk	Qom	11.6
MG102	Goat	Milk	Qom	28.18
MG103	Goat	Milk	Qom	31.44
AG1	Goat	Aborted fetal fluids	Tehran	17.98
AG2	Goat	Aborted fetal fluids	Tehran	29.08
AG3	Goat	Aborted fetal fluids	Tehran	26.16
AG4	Goat	Aborted fetal fluids	Mahabad	31.72
MS16	Sheep	Milk	Babol	32.78
MS45	Sheep	Milk	Qom	25.01
MS46	Sheep	Milk	Qom	27.99
MS51	Sheep	Milk	Qom	25.46
MS52	Sheep	Milk	Qom	30.62
MS64	Sheep	Milk	Hamadan	31.79
AS3	Sheep	Aborted fetal fluids	Tehran	25.5
AS25	Sheep	Spleen aborted fetus	Urmia	25.91
AS26	Sheep	Spleen aborted fetus	Urmia	26.13
AS43	Sheep	Cotylodon	Hamadan	30.16
Q34	Human	Cardiac valve	Tehran	34.2

*Quantification cycle (Cq).

### MLVA/VNTR capillary electrophoresis genotyping

Since six known loci or VNTRs, namely ms23, ms24, ms27, ms28, ms33, and ms34, were utilized for genotyping *C. burnetii* using capillary electrophoresis in established *C. burnetii* MLVA/VNTR studies, we also initially targeted these six loci with the previously designated primers^22,24^. However, during gel electrophoresis of the amplified ms23 locus, an unusual fragment with a length of 1500 bp was observed in all 26 samples analyzed. Sequencing analysis of the PCR product from this locus (ms23) for all samples revealed that the IS1111 insertion sequence was present at this site, causing an increase in its size. Therefore, the amplification of the ms23 locus was unsuccessful for the MLVA/VNTR study. Considering this limitation, we excluded it from the analysis and utilized five loci instead of six. Reverse primers for the ms23, ms33, and ms34 loci were labeled with the 6-FAM fluorescent color, while reverse primers for the ms24, ms27, and ms28 loci were labeled unilaterally with carboxy 4, 7, 2', 4', 5', 7' hexachloro fluorescein (HEX) -6. The PCR reaction was performed using the commercial TEMPase Hot Start 2× Master Mix BLUE (Ampliqon, Odense M, Denmark). Each reaction contained 5 μl of extracted DNA, 12.5 μl of Mastermix, 400 mmol of each primer, and distilled water added to achieve a final volume of 25 μl. The standard strain used in this study was the Nine Mile RSA493 *C. burnetii* strain.

The PCR temperature program consisted of an initial denaturation step at 95 °C for 15 min, followed by 40 cycles of denaturation at 94 °C for 30 s, annealing at 60 °C for 60 s, extension at 72 °C for 1 min, and a final extension step at 72 °C for 10 min. To confirm the presence of the desired band and the absence of non-specific bands, the PCR product for each locus was separated by size using a 2% agarose gel. Following successful amplification, capillary electrophoresis was carried out to determine the exact size of each locus. The purified PCR products were obtained using a PCR product purification kit (Kawsar biotech, Tehran, Iran) and were further diluted at ratios of 1: 5, 1: 10, and 1: 100. Then, 2 μl of each diluted sample was added to a solution containing 10.5 μl of formide and 0.5 μl of standard marker size 560 (Applied Biosystems, Foster City, USA). The mixture was thoroughly mixed, denatured at 95 °C for 30 s, and then placed on ice for 2 min. Finally, the samples were run on a Genetic Analyzer capillary sequencer model 3130x (Applied Biosystems, Foster City, USA) using POP7 polymer.

The data obtained from capillary electrophoresis were analyzed using GeneMarker software, version 1.6 (Pennsylvania, USA), regarding the standard marker size and the fluorescent dye for each locus.

### Determine the number of repeats for each VNTR locus

The number of repeats for each VNTR locus was determined by comparing the sizes of the fragments obtained with those obtained from the reference strain Nine Mile RSA 493 in the same run. Based on in silico analysis, the established genotype of the strain was found to have 9, 27, 4, 6, 9, and 5 repeats for marker loci Ms23, Ms24, Ms-27, Ms28, Ms33, and Ms34, respectively.

The following formula, which replaces the size and number of repeats in each locus of the standard Nine Mile RSA493 *C. burnetii* strain, was used to determine the number of repetitions for each VNTR locus in the samples^25^.

$${\text{Number}}\,{\text{of}}\,{\text{Repeats}} = \frac{{{\text{Amplicon}}\,{\text{Size}}\,{\text{of}}\,{\text{sample}}_{{({\text{bp}})}} - {\text{Amplicon}}\,{\text{Size}}\,{\text{in}}\,{\text{RSA}}\,{493}_{{({\text{bp}})}} }}{{{\text{Repeat}}\,{\text{Size}}_{{({\text{bp}})}} }} + {\text{Number}}\,{\text{of}}\,{\text{Repeats}}\,{\text{in}}\,{\text{RSA}}\,{493}$$

### VNTR data analysis

The discriminatory power of each selected VNTR locus was calculated by the Hunter and Gaston discriminatory index (HGDI)^26^.

The MLVA/VNTR data of the examined samples were analyzed using the online MLVAbank for Microbes Genotyping database (http://microbesgenotyping.i2bc.paris-saclay.fr/). The profiles of each sample were compared with previously registered strains in the database. Genotypes that were not previously registered in the database were considered new genotypes of *C. burnetii*. The data from five capillary loci in this study were compared against the information available in the databases.

The phylogenetic analysis was conducted using the advanced Bionumerics 6.6 software package (Applied Maths, Sint-Martens-Latem, Belgium). The comparison of the position of *C. burnetii* strains with other data recorded in the database was determined using the Unweighted Pair Group Method with Arithmetic Mean (UPGMA) and Minimum Spanning Tree algorithms. Genomic grouping for *C. burnetii* was performed using cluster analysis for MLVA/VNTR capillary electrophoresis data.

### Ethics

This study, including the proposal, all experimental protocols, and consent procedure, was approved by the Ethics Committee for Biomedical Research of Tarbiat Modares University (Ethic Code: IR.TMU.REC.1395.510). All methods were carried out in accordance with the relevant guidelines and regulations of the Ethics Committee for Biomedical Research of Tarbiat Modares University. The related methods are reported following the ARRIVE guidelines (https://arriveguidelines.org).

We used the samples available in our biobank, which were collected during our previous studies^10,19–21^. Therefore, the requirement for an informed consent statement was waived.

## Results

### MLVA/VNTR genotyping with the five-locus panel using capillary electrophoresis

**Figure 1:**
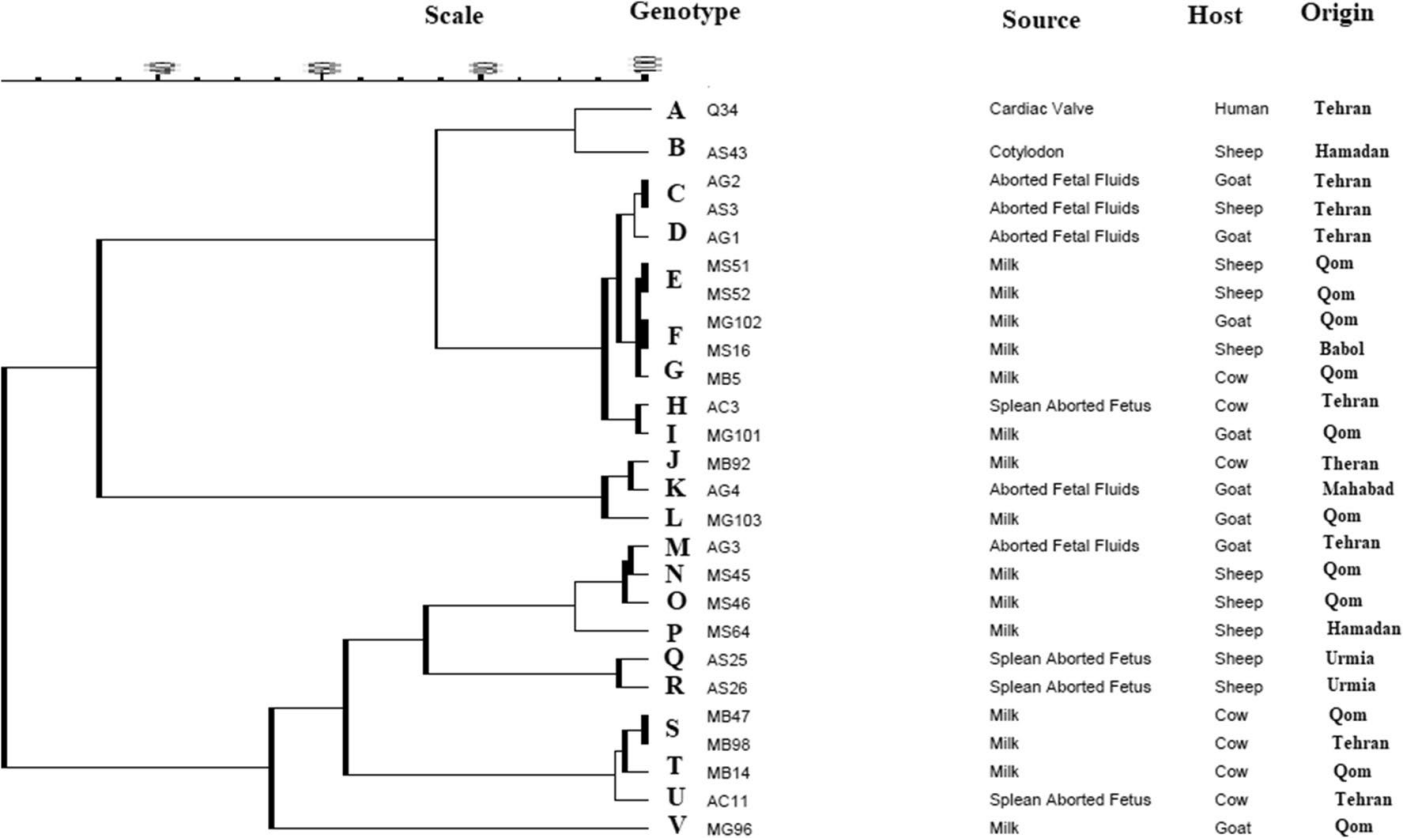
Dendogram designed of *Coxiella burnetii* based on UPGMA analysis of MLVA/VNTR 5 loci (ms24, ms27, ms28, ms33 and ms34).

Finally, 22 genotypes (A to V) of *C. burnetii* were observed among the studied samples in this study (Table 2). The genotype of the human heart valve sample (Sample Q34) was relatively determined as it was amplified in only three out of the five loci tested. However, it showed the closest similarity to the standard control strain (Nine Mile RSA493 *C. burnetii*). Furthermore, genotypes C (samples AG2 and AS3), E (samples MS51 and MS52), F (samples MG103 and MS16), and S (samples 9898 and MB47) included two samples with similar allelic profiles. Based on genotyping results of 27 samples for the panel of five tested loci, the overall HGDI value of the MLVA/VNTR genotyping method was estimated at 0.740. The HGDI value for a single locus ranged from 0.493 to 0.865. The HGDI value for ms24 (10 alleles), ms27(5 alleles), ms28(8 alleles), ms33 (10 alleles), and ms34 (8 alleles) was 0.836, 0.493, 0.729, 0.752, and 0.865, respectively. The UPGMA analyses of samples in this study are presented in Fig. 1.

**Table 2:** Allelic profiles of different *Coxiella burnetii* strains based on MLVA/VNTR Capillary electrophoresis Genotyping.

Sample ID	Host	Origin	ms24	ms27	ms28	ms33	ms34	MLVA type
MB5	Cattle	Qom	12	3	3	7	9	**G**
MB14	Cattle	Qom	1	4	4	27	9	**T**
MB47	Cattle	Qom	3	4	4	7	4	**S**
MB92	Cattle	Tehran	13	14	7	9	10	**J**
MB98	Cattle	Tehran	3	4	4	7	4	**S**
AC3	Cattle	Tehran	12	4	3	7	7	**H**
AC11	Cattle	Tehran	3	4	4	7	5	**U**
MG96	Goat	Qom	10	14	8	16	2	**V**
MG101	Goat	Qom	14	4	3	7	7	**I**
MG102	Goat	Qom	11	4	3	7	9	**F**
MG103	Goat	Qom	11	14	3	8	9	**L**
AG1	Goat	Tehran	11	4	2	8	8	**D**
AG2	Goat	Tehran	12	4	3	8	8	**C**
AG3	Goat	Tehran	12	4	3	20	7	**M**
AG4	Goat	Mahabad	14	14	2	7	7	**K**
MS16	Sheep	Babol	11	4	3	7	9	**F**
MS45	Sheep	Qom	11	4	3	25	7	**N**
MS46	Sheep	Qom	11	3	ND	23	2	**O**
MS51	Sheep	Qom	12	4	3	7	9	**E**
MS52	Sheep	Qom	12	4	3	7	9	**E**
MS64	Sheep	Hamadan	12	9	3	22	2	**P**
AS3	Sheep	Tehran	12	4	3	8	8	**C**
AS25	Sheep	Urmia	6	4	6	7	4	**Q**
AS26	Sheep	Urmia	6	4	6	7	3	**R**
AS43	Sheep	Hamadan	8	4	5	6	2	**B**
Q34	Human	Tehran	ND	2	23	9	ND	**A**
RSA493	Tick	USA	27	4	6	9	5	**Ref strain**

ND: not determined.

**Figure 2:**
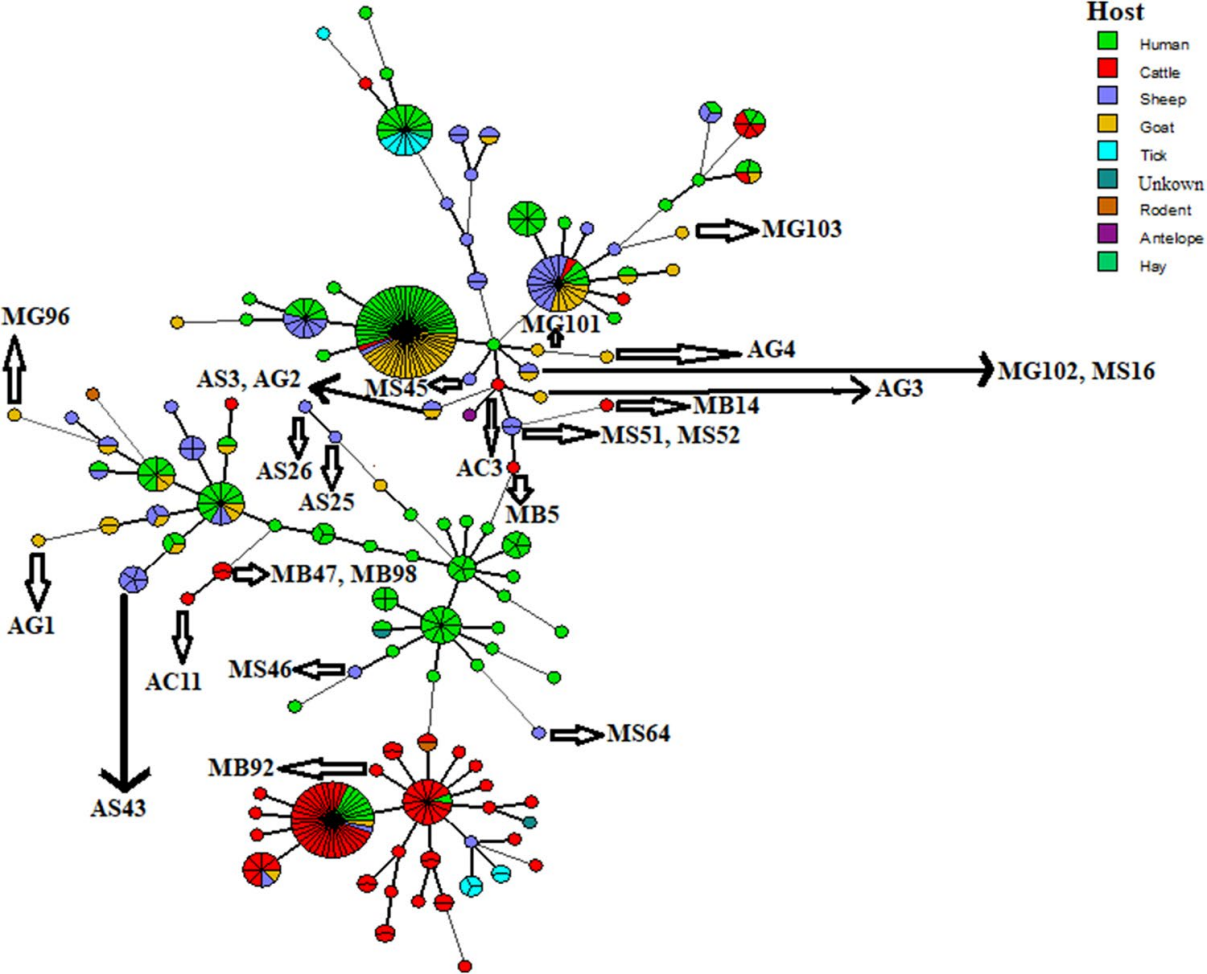
MLVA/VNTR analysis of *Coxiella burnetii* using the Minimum Spanning Tree algorithm for MLVA/VNTR 5 loci (ms24, ms27, ms28, ms33 and ms34) data obtained from the online MLVAbank for Microbes Genotyping database (http://microbesgenotyping.i2bc.paris-saclay.fr/). The origin of the samples is represented by different colors. Our samples are indicated with an arrow.

The Minimum Spanning Tree algorithm analysis of *C. burnetii* samples in Iran, compared to world strains (based on data recorded in the online MLVAbank for Microbes Genotyping database), is shown in Fig. 2.

### In silico cluster analysis

**Figure 3:**
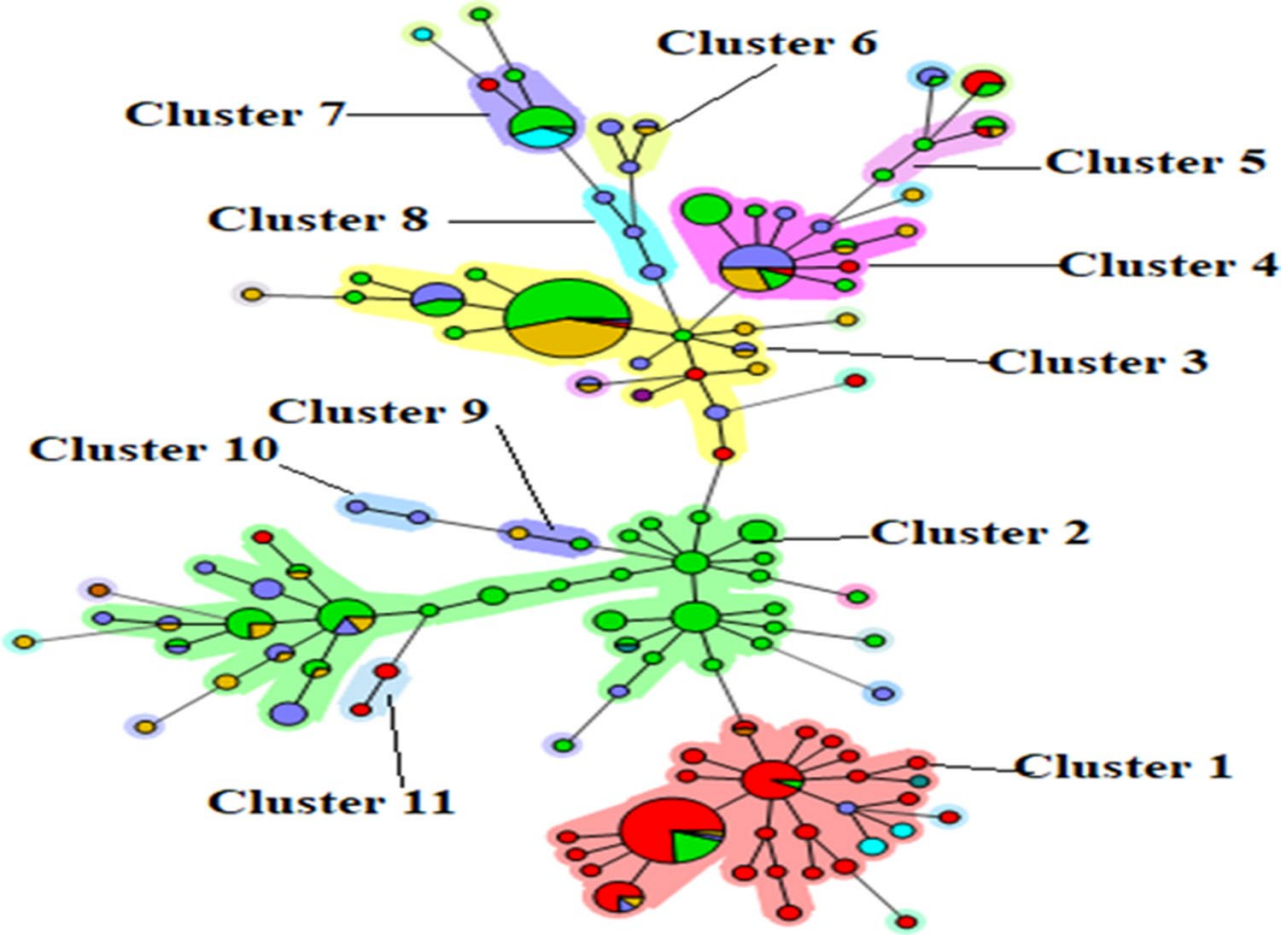
Genomic grouping (cluster 1–11) of *Coxiella burnetii* using the Minimum Spanning Tree algorithm based on data obtained from the online MLVAbank for Microbes Genotyping database (http://microbesgenotyping.i2bc.paris-saclay.fr/)and the clustering of genotypes.

Using cluster analysis of MLVA/VNTR capillary electrophoresis data, 11 genomic and clonal complex groups were identified for *C. burnetii* (Fig. 3). Iranian *C. burnetii* strains were categorized into five distinct genomic groups (clusters 1, 2, 3, 10, and 11) as well as seven singletons. The majority of Iranian samples (nine strains) were classified in cluster 3. Clusters 11 (three samples) and 10 (two samples) exclusively consisted of Iranian samples. Cluster 2 represented the largest genome group, with two samples from Iran. Additionally, cluster 1, the second-largest cluster, featured a sample (MB92) from Iran.

## Discussion

Q fever has emerged as a significant global public health concern due to various factors, including the wide range of hosts that *C. burnetii* can infect, its transmission through aerosols, its low infectious dose, and its ability to survive in the environment for long periods. Early studies demonstrated that *C. burnetii* strains collected from different regions and sources exhibit differences in virulence and signs of disease, which can be attributed to the diversity of *C. burnetii* genotypes^17,27,28^. Therefore, genotyping provides valuable insights into the genotype of circulating *C. burnetii* strains and helps to identify sources. These capabilities make genotyping an essential tool for the control of *C. burnetii* outbreaks^29^. The present study reported the first genotyping of *C. burnetii* strains in Iran using the MLVA/VNTR method with five different loci (Ms24, Ms27, Ms28, Ms33, and Ms34) and capillary electrophoresis. Unfortunately, one locus (Ms23) was excluded from genotyping due to the non-successful amplification caused by an IS1111 insertion inside it. This insertion has also been reported in some strains of *C. burnetii* in previous studies^30,31^. In addition, out of the five loci tested, only three were amplified in sample Q34 (human heart valve), resulting in a partially determined genotype. The genotype of this sample was relatively close to the cotyledon sample derived from a sheep (AS43).

Using this genotyping panel, we identified 22 different genotypes (A to V) among the 26 livestock and human samples selected from six cities, indicating a high level of diversity in genotyping *C. burnetii* genotypes in Iran. This is not surprising, as previous studies have also shown a diverse range of genotypes associated with different hosts or sources of *C. burnetii* infection^17,27,28^.

Among the samples derived from sheep, goats, and cattle, we identified nine, eight, and six different genotypes, respectively. However, two genotypes (F and C) were shared between samples from sheep and goats, suggesting some commonality in the genotypes of *C. burnetii* infecting these host species. Several studies conducted in multiple countries have discovered various MLVA/VNTR genotypes of *C. burnetii* that infect dairy cattle herds^29,32,33^. For example, in Germany, 32 distinct genotypes were identified among 104 *C. burnetii* strains derived from cattle and sheep^34^. In Italy, 23 different genotypes were reported among 34 *C. burnetii* isolates infecting dairy cattle products^35^. Similar diversity was observed in *C. burnetii* strains isolated from human clinical and animal samples in other countries like Portugal^36^ and Hungary^37^. In the Netherlands, identical genotypes between humans and goats showed that goats were likely sources of human infection^38^. These studies highlight the diverse genotypic variations of *C. burnetii* isolates across different hosts and regions, emphasizing the importance of comprehensive genotyping methods in understanding the epidemiology of this pathogen. In addition to host-related genetic diversity, we also observed genetic diversity based on geographic distribution within Iran. Each city in the present study had different genotypes, except for two in Qom (genotype E) and two in Tehran (genotype C). The genotypes identified in this study were specific to different regions and the country. These variations may be influenced by different climate and environmental conditions in each region, as the epidemiology of tick-borne diseases has been shown to be significantly affected by environmental changes resulting from both the climate emergency and human activities^39^.

The cluster analysis of available *C. burnetii* MLVA/VNTR capillary electrophoresis data revealed the presence of 11 (1–11) genomic and clonal complex groups recorded in the word. Furthermore, this analysis revealed that *C. burnetii* strains in Iran were classified into five different genomic groups, with two clusters (10 and 11) exclusively comprised of strains from Iran. These findings highlight the distinctiveness of *C. burnetii* genotypes in Iran compared to those reported from other parts of the world. However, the limitations of this study include the relatively small sample size, the inability to amplify and analyze the ms23 locus (this reduced the MLVA/VNTR panel to 5 loci instead of 6, limiting resolution), and the lower resolution offered by MLVA compared to other advanced genotyping techniques like whole-genome sequencing. Future studies with larger sample sizes and more comprehensive genotyping methods are needed to further enhance our understanding of the molecular epidemiology of *C. burnetii* in Iran.In conclusion, the strains described in the present study, exhibiting various host tropisms and geographical origins, have revealed the diverse genetic profiles of *C. burnetii* strains in Iran. Genotyping analysis revealed significant differences between circulating strains of *C. burnetii* in Iran compared to strains from other regions. This information provides valuable insights into the molecular epidemiology of *C. burnetii* in Iran and emphasizes the need for comprehensive surveillance, prevention, and treatment strategies for Q fever in the country.

## Data Availability

Data supporting the findings of this study are available within the article and can be obtained from the corresponding author upon request. The MLVA/VNTR genotyping data for *C. burnetii* in the present study have been deposited in MLVAbank for Microbes Genotyping (https://microbesgenotyping.i2bc.paris-saclay.fr/).
